# The Cancer Wards at the Middlesex Hospital

**Published:** 1900-04-28

**Authors:** 


### Editor's Letter-Box.

[Our Correspondents are reminded that prolixity is a great bar to publication, and that brevity of style and conciseness of statement greatly facilitate early insertion.]

### THE CANCER WARDS AT THE MIDDLESEX HOSPITAL.

Mr. Keith D. Young, F.R.I.B.A., writes: "I have only just seen the notice of the new cancer wards of the Middlesex Hospital in your issue of April 7th. Will you permit me to explain two points? With reference to the curved bay window on first and second floors, you say that by projecting the porch a few feet further out we could have had a deeper bay. This is very true, but at the same time it would not have been possible to have built the porch further out, and if it had been, it is more than doubtful whether the County Council would have consented to any further projection; the bay itself is a projection beyond the general frontage line, and in order to build it at all we had to apply for special permission to the Council. With regard to the operation-room and suggestion of a top light, in the first place the room was originally intended as a ward and not as an operation-room; and secondly, the surgeons are perfectly contented with the light as it is."

